# Supplementary material for: Integrated analysis of the aqueous humor microbiome and lens capsule transcriptome in high myopia cataract: a pilot study
Source: Front Med (Lausanne). 2026 Jun 16;13:1845205. doi: 10.3389/fmed.2026.1845205 (PMC13314463; doi:10.3389/fmed.2026.1845205)
Supplement: Supplementary file 3 [file Data_Sheet_1.zip › 4.Meta_Statistic/C372089/T_test/Genus/Genus.T_test.diff.heatmap.pdf]

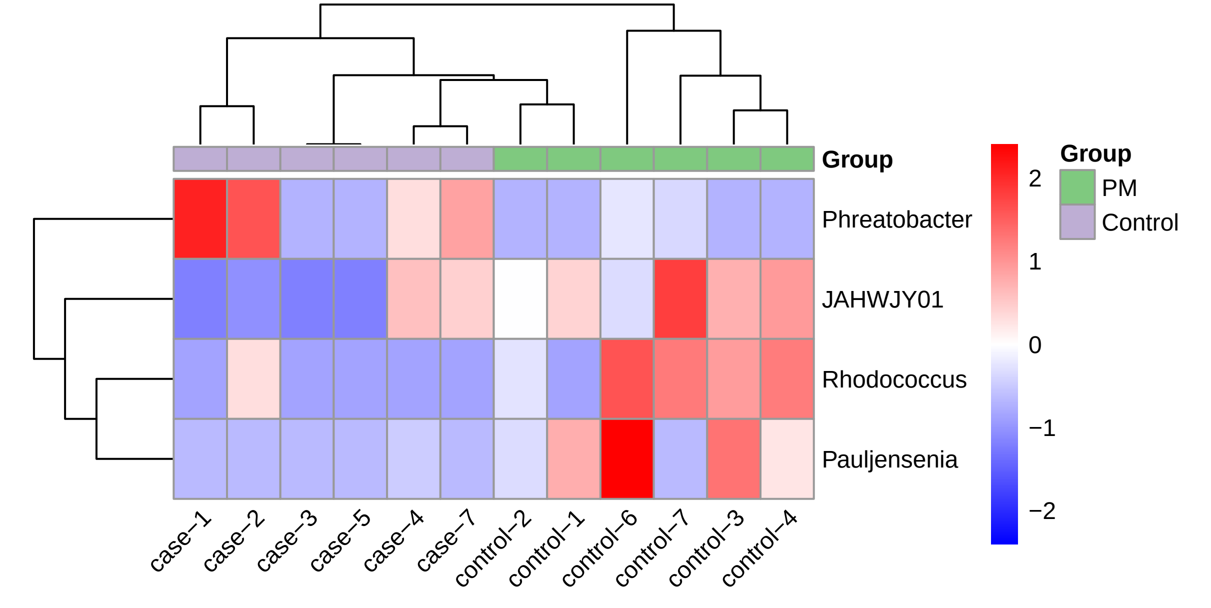

Group
Phreatobacter
JAHWJY01
Rhodococcus
Pauljensenia
case−1
case−2
case−3
case−5
case−4
case−7
control−2
control−1
control−6
control−7
control−3
control−4
2
1
0
−1
−2
Group
PM
Control
